# Outcomes of 4819 cases of marine animals presented to a wildlife rehabilitation center in New Jersey, USA (1976–2016)

**DOI:** 10.1038/s41598-021-81634-5

**Published:** 2021-01-26

**Authors:** Stefan H. Gallini, Nicola Di Girolamo, Elizabeth Hann, Hubert Paluch, Peter M. DiGeronimo

**Affiliations:** 1 Department of Clinical Sciences and Advanced Medicine, School of Veterinary Medicine, University of Pennsylvania, Philadelphia, PA 19104 USA; 2 Department of Veterinary Clinical Sciences, College of Veterinary Medicine, Oklahoma State University, Stillwater, OK 74078 USA; 3 Adventure Aquarium, Camden, NJ 08103 USA; 4 Marine Mammal Stranding Center, Brigantine, NJ 08203 USA; 5 Present Address: Animal and Bird Health Care Center, Cherry Hill, NJ 08003 USA

**Keywords:** Conservation biology, Population dynamics

## Abstract

Understanding marine animal stranding patterns can aid rehabilitation efforts and evaluations of ecosystem health. The goal of this retrospective study was to identify factors associated with outcome of marine animals presented to a rehabilitation facility in Brigantine, New Jersey, USA. Records of 4819 phocids, cetaceans, and sea turtles were reviewed. Taxa, age, sex, season, and outcome (natural death, euthanasia, transfer to another facility, and successful release) were recorded for each case. Binary logistic regression was employed to identify predictors associated with release, and a multivariate logistic regression model was developed to evaluate whether the association between taxa and chance of release persisted after adjustment for the other variables. Phocids were most likely to strand during winter. Phocids and sea turtles that stranded alive were more likely to be released than to die under care or be euthanized. Taxa, age, and season were all significantly associated with the probability of release. These results provide a reference for phocid, cetacean, and sea turtle stranding and rehabilitation in part of the mid-Atlantic region. Critical evaluation of wildlife rehabilitation is indicated to audit the success of efforts and to assess threats to free-ranging populations.

## Introduction

Marine animals such as pinnipeds, cetaceans, and sea turtles are charismatic megavertebrates that garner much attention from the public. Relatively long lifespans, exposure to biological and chemical insults in ocean ecosystems, and, for marine mammals, occupation of high trophic levels allow these species to serve as indicators of ecosystem health^1,2^. Species that inhabit coastal waters serve as sentinels of ocean health because of their exposure to anthropogenic pressures on marine ecosystems including terrestrial effluents, boat traffic, light and noise pollution, and other effects of coastal land use and maritime industries such as fisheries^3–7^. Species that inhabit pelagic habitats can similarly serve as sentinels due to exposure to anthropogenic threats including oil spills and bycatch^8,9^.

With the exceptions of pinnipeds hauled out on shore and nesting sea turtles, sampling marine animal populations is difficult due to the cryptic nature of their underwater and offshore coastal or pelagic habitats. Marine animals that are found on beaches and unable to return to the water unassisted are considered stranded^10,11^ and when found are those that are presented for potential rehabilitation, if alive, or for post-mortem examination, if dead. Presentation of wild animals to rehabilitation centers can serve as passive and opportunistic, albeit biased, sampling of free-ranging populations^12^. Although wildlife rehabilitators may have the most direct contact with wild animals of any group of stakeholders, rehabilitation records are infrequently used for scientific assessment of free-ranging populations often due to variability in data collection, inconsistency of medical records, inherent biases in the sample population, and reluctance to collaborate with researchers outside of the organization^12^. Despite these difficulties, retrospective data from marine animal rehabilitation centers have been used to identify anthropogenic causes of stranding, demonstrate population level effects of stranding events, and allow for evidence-based strategies to mitigate such effects^13–18^. Retrospective studies of wildlife morbidity and mortality rehabilitation records can provide “insight into the life history of species involved, their habitat, baseline health parameters, and anthropogenic factors that may affect their survival”^3^. Furthermore, such studies serve as an audit of rehabilitation centers, elucidating the relative success and outcomes of rehabilitation efforts^19^. The purpose of this retrospective study was to use records of stranded marine animals (phocids, cetaceans, and sea turtles) recovered by a rehabilitation center in New Jersey, USA to assess for temporal patterns of stranding and the likelihood of release between taxa and to identify factors associated with release of stranded animals.

## Materials and methods

All records reviewed over the entire course of the study period (1976–2016) came from the Marine Mammal Stranding Center (MMSC) located in Brigantine, New Jersey, USA. The MMSC is the sole responder to both live and dead strandings of phocids, cetaceans, and sea turtles on the New Jersey coast since 1976 and represents the entire New Jersey coast within the Greater Atlantic Stranding Network. All stranding events to which the MMSC responds are reported through passive surveillance by bystanders or local municipalities.

The rehabilitation facilities of the MMSC are used primarily for pinnipeds and sea turtles and consist of a series of small to mid-sized holding tanks and a large pool. Since the inception of the center, represented in the beginning of the study period, the capacity of the MMSC to respond to stranding events has expanded with the construction of additional holding facilities, increased staffing and funding, and the addition of improved transport vehicles and equipment.

### Case definition

All cases accessioned by the MMSC from January 1976 through December 2016 were available for review. The species, sex, age class, date of intake, and final disposition of each case was recorded. Sex was categorized as male, female, or undetermined and age class as neonatal, juvenile, or adult based on external anatomy and morphology. Dates of intake were categorized by month and by season. Seasons were defined as winter (21 December through 20 March), spring (21 March through 20 June), summer (21 June through 20 September), and autumn (21 September through 20 December). Final disposition included those animals that were successfully released to natural habitat and those that were not released. Animals not released included those that were deceased upon arrival of MMSC staff, those that died naturally while under care, those that were euthanized, and those that were deemed unsuitable for release and transferred to another facility. Each case that presented alive to MMSC responders received care either on the beach stranding site or at the rehabilitation center. Descriptive and analytic statistics were obtained using a spreadsheet (Excel, version 15.34.0, Microsoft Corp, Redmond, WA) and a statistical software package (SPSS statistics 24.0, IBM, Chicago, IL).

### Temporal patterns of stranding between taxa

To investigate potential temporal patterns of stranding between taxa (i.e. phocids, cetaceans, and sea turtles), multinomial logistic regressions were performed with taxa as the dependent variable and season and month of intake as independent variables. Most animals that were presented alive were phocids, so this taxon was used as the reference.

### Likelihoods of death and euthanasia between taxa

To investigate potential differences in outcomes between taxa for those animals that were presented alive, multinomial logistic regressions were performed after exclusion of animals that were found dead at time of accession. Final disposition was set as the dependent variable and taxa as the independent and the regression repeated with death while under care and euthanasia used as references. Odds ratios (OR) and 95 percent confidence intervals (95% CI) were used to quantify the strength of all associations.

### Predictors associated with release of stranded animals

Continuous variables were summarized as either mean and standard deviation or median and interquartile ranges depending on their distribution. The Shapiro–Wilk test was used to analyze continuous data for normality. For categorical variables, the percentages of animals in each category were calculated.

Binary logistic regression was employed in order to identify predictors associated with release of the stranded animal. At first, univariate logistic regression models were built with each of the following predictors: taxa [phocid/cetacean/chelonian]; age [adult/juvenile/neonate]; sex [male/female/unknown]; season [winter/spring/summer/autumn]; decade [1976–1986/1987–1996/1997–2006/2007–2016]. Odds ratios and 95 percent confidence intervals were used to quantify the strength of these associations.

Multivariate logistic regression models were subsequently developed to evaluate whether the association between taxa and chance of being released persisted after adjustment for the other variables. Patient characteristics and covariates demonstrated in the univariate analysis to be associated with released chance at a significance level of *P* < 0.05 were entered into the final model. The Hosmer–Lemeshow statistic was used in order to assess goodness-of-fit of the model. Standard multicollinearity diagnostics for logistic regression models were employed, using a threshold value for condition indices > 30^20^. The Nagelkerke R^2^ was reported as a measure of predictive power of the model. To avoid overfitting the model no more than 1 predictor variable was included for 10 events (i.e. animals successfully released)^20^.

## Results

### Descriptive statistics

Between 1976 and 2016, 4,819 cases were accessioned by the Marine Mammal Stranding Center. Of these, 1,478 (30.7%) were phocids, 1,408 (29.2%) were cetaceans, and 1,926 (40.0%) were sea turtles. The remaining cases included 3 sirenians (2 Antillean manatees [*Trichechus manatus manatus*] and 1 Florida manatee [*Trichechus manatus latirostris*]), 1 otariid (a California sea lion [*Zalophus californianus*]), 1 partial carcass presumed to be of an elasmobranch (basking shark [*Cetorhinus maximus*]), and 2 carcasses of unidentified taxa, all of which were excluded from subsequent statistical analyses. The majority of cases (98.6%; 4753/4819) were from New Jersey with the remainder of cases recovered or transferred from 7 other states (Table 1). Cases were seen in all months and every season of each year.

**Table 1:** Number of cases presented to the Marine Mammal Stranding Center according to state of origin between 1976 and 2016.

State	*N*	Percentage
New Jersey	4753	98.6%
Pennsylvania	7	0.15%
Delaware	22	0.46%
Maryland	9	0.19%
North Carolina	10	0.21%
Virginia	11	0.23%
Florida	1	0.02%
Maine	6	0.12%

**Figure 1:**
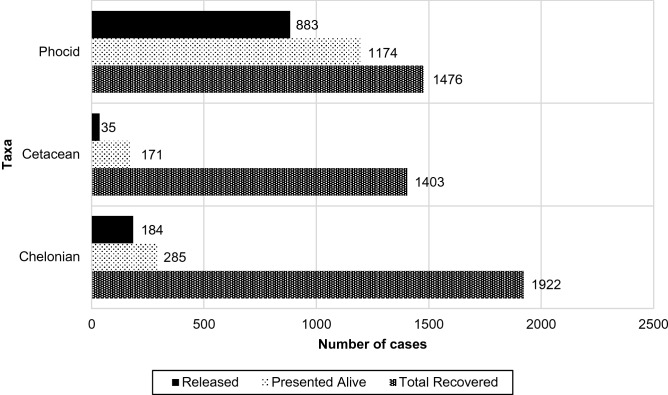
Number of cases with known outcomes per taxa of animals presented to the Marine Mammal Stranding Center in Brigantine, NJ between 1976 and 2016. Bars represented to the total number of animals presented, the proportion alive at presentation, and the proportion of live stranded animals that were released to their natural habitat.

Of the 4,801 animals for which final outcomes were known, 79.54% (1174/1476) phocids, 12.19% (171/1403) cetaceans, and 14.83% (285/1922) sea turtles were presented alive. Of these, 75.21% (883/1174) phocids, 20.47% (35/171) cetaceans, and 64.56% (184/285) sea turtles were released (Fig. 1).

Across all 1,630 cases that presented alive and where age, final disposition, and date of presentation were known, 73.5% (982/1336) of juvenile animals, 41.3% (118/286) of adult animals, and 25.0% (2/8) of neonatal animals were released. Of all animals presented during each decade, 33.3% (17/51) of animals presented from 1976–1986, 70.2% (229/326) of animals presented from 1987–1996, 68.9% (371/538) of animals presented from 1997–2006, and 67.8% (485/715) of animals presented from 2007–2016 were released. Of all animals presented, 72.4% (580/801) that presented in winter, 68.7% (241/351) from the spring, 59.5% (175/294) from summer, and 57.6% (106/184) from autumn were released. Of all animals presented, 27.3% (370/576) of females, 67.5% (529/784) of males, and 75.2% (203/270) of those of unknown sex were released.

### Temporal patterns of stranding between taxa

**Figure 2:**
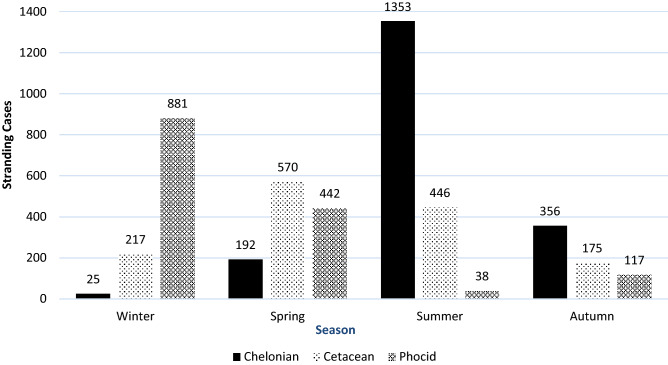
Total number of cases of chelonians, cetaceans and phocids presented the Marine Mammal Stranding Center between 1976 and 2016 according to season.

More phocids stranded in winter (n = 881) and spring (n = 442) than in summer (n = 38) and autumn (n = 117). The presentation of cetaceans was more evenly distributed throughout all seasons, although more were presented in spring and summer than in autumn or winter (570 in spring, 446 in summer, 175 in autumn, and 217 in winter), Sea turtles had an inverse seasonal distribution with more presented in summer (n = 1,353) and autumn (n = 356) than in winter (n = 25) or spring (n = 192) (Fig. 2).

Cetaceans were more likely to strand in spring (OR 1.290, 95% CI 1.14 to 1.46; *P* < 0.0001), summer (OR 11.436, 95% CI 8.24 to 15.87; *P* < 0.0001), and autumn (OR 1.496, 95% CI *P* = 0.001) and less like to strand in winter (OR 0.246, 95% 0.21 to 0.29; *P* < 0.0001) compared to phocids. Compared to phocids, sea turtles were over 34 times more likely to be presented in summer (OR 34.692, 95% CI 25.23 to 47.70; *P* < 0.0001) and 3 times more likely to be presented in autumn (OR 3.043, 95% CI 2.47 to 3.75; *P* < 0.0001). Sea turtles were significantly less likely to be presented in winter (OR 0.028, 95% 0.02 to 0.04; *P* < 0.0001) and spring (OR 0.043, 95% 0.37 to 0.52; *P* < 0.0001) compared to phocids.

Relative to phocids, cetaceans were substantially more likely to be presented in May through October and less likely to be presented in January through April. There was no considerable difference in likelihood of presentation in November and December. Sea turtles were more likely than phocids to be presented in June through October and less likely to be presented in December through May. There was no considerable difference in likelihood of presentation in November.

### Likelihoods of death and euthanasia between taxa

Of all animals that were alive at presentation, phocids were over 4 times more likely to be released than to die under care (OR 4.340, 95% CI 3.730 to 5.050; *P* < 0.0001) but were less likely to be euthanized (OR 0.243, 95% CI 0.178 to 0.331; *P* = 0.020) or transferred to another facility (OR 0.173, 95% CI 0.154 to 0.296; *P* < 0.0001). Cetaceans were less likely to be euthanized (OR 0.653, 95% CI 0.456 to 0.936; *P* = 0.020), to be released (OR 0.507, 95% CI 0.343 to 0.749; *P* = 0.001), or to be transferred (OR 0.173, 95% CI 0.096 to 0.312; *P* < 0.0001) than to die while under care. Sea turtles were over 5 times more likely to be released than to die under care (OR 5.667, 95% CI 3.914 to 8.204; *P* < 0.0001). There were no significant differences between the likelihoods of a sea turtle being transferred to another facility (*P* = 0.413) or being euthanized (*P* = 0.706) and dying while under care.

Of all animals that were alive at presentation, phocids were over 17 times more likely to be released (OR 17.880, 95% CI 13.448 to 23.772; *P* < 0.0001) and over 4 times more likely to die under care (OR 4.120, 95% CI 3.025 to 5.612; *P* < 0.0001) than to be euthanized. There was no significant difference between the likelihood of a phocid being transferred to another facility and being euthanized (*P* = 0.234). Cetaceans were more likely to die under care (OR 1.531, 95% CI 1.068 to 2.194; *P* = 0.020) and less likely to be transferred (OR 0.265, 95% CI 0.144 to 0.489; *P* < 0.0001) than to be euthanized. There was no significant difference between the likelihood of a cetacean being released and being euthanized (*P* = 0.240). Sea turtles were more than 6 times more likely to be released (OR 6.233, 95% CI 4.239 to 9.165; *P* < 0.0001) than to be euthanized. There were no significant differences between the likelihoods of a sea turtle being transferred to another facility (*P* = 0.234) or dying under care (*P* = 0.706) and being euthanized.

### Predictors associated with release of stranded animals

In the univariate regression models, taxa [phocid/cetacean/chelonian]; sex [male/female/unknown]; season [winter/spring/summer/autumn]; and decade [1976–1986/1987–1996/1997–2006/2007–2016] were all associated with a difference in release chance. A multivariate regression model including “taxa”, “age”, “sex”, “season” and “decade” was fitted (Hosmer–Lemeshow test *P* = 0.382) and the model explained 22% of the variability of the outcome (Nagelkerke R^2^ = 0.22).

Phocids were the stranded animals most likely to be released, with cetaceans and chelonians having approximately 17 times (OR 17.55, 95% CI 9.57 to 32.21; *P* < 0.0001) and 8 times (OR 7.85, 95% CI 4.32 to 14.26; *P* < 0.0001) the odds of not being released, respectively. Although more juvenile animals were released than neonates or adults, the effect was not statistically significantly different (odds of neonates not being released OR 4.79, 95% CI 0.81 to 28.22; *P* = 0.084; odds of adults not being released OR 1.10, 95% CI 0.73 to 1.67; *P* = 0.638). Animals for which sex was determined were about 6 times more likely to not be released (OR for females: 6.70, 95% CI 4.12 to 10.90; *P* < 0.0001; OR for males: 6.40, 95% CI 3.86 to 10.61; *P* < 0.0001) than animals for which sex was not determined. Animals stranded during winter (OR 0.89, 95% CI 0.59 to 1.35; *P* = 0.593) and spring (OR 0.77, 95% CI 0.50 to 1.20; *P* = 0.248) had equal likelihoods of release as those stranded during autumn, whereas animals stranded during summer had greater odds of being released (OR 0.59, 95% CI 0.36 to 0.96; *P* = 0.033). Animals that were presented to the center during the first decade (1976–1986) were twice as likely not to be released as animals in the most recent decade (2007–2016) (OR 2.03, 95% CI 1.01 to 4.06; *P* = 0.046). The odds of release were not significantly different for animals presented from 1987 through 1996 (OR 0.83, 95% CI 0.60 to 1.14; *P* = 0.243) or from 1997 through 2006 (OR 0.90, 95% CI 0.69 to 1.18; *P* = 0.450) than for those presented from 2007 through 2016.

## Discussion

Through retrospective analysis, wildlife rehabilitation records can be used to elucidate temporal patterns of morbidity and mortality in free-ranging populations and can critically evaluate the likelihood of outcomes of clinical intervention^19^. Future investigations are indicated to determine prognostic indicators of successful release and to assess survival of rehabilitated animals to evaluate long-term success. Data collected by the MMSC contributes to the Greater Atlantic Marine Mammal Stranding Network database and provides valuable information used to assess the health of larger, regional populations. Cases were roughly evenly distributed between phocids, cetaceans and sea turtles, all native to NJ coastal waters. The three sirenians reported were anomalous, having been recovered north of their typical range^21^ presumably following warm water currents during summer and autumn months. The single California sea lion presented to the center belonged to a U.S. Navy base and was not a free-ranging animal.

Sampling bias is a significant limitation of studies of free-ranging wildlife that rely on opportunistic stranding or presentation for rehabilitation^3^. Factors such as the cause, location, or season of stranding may contribute to sampling bias. Pinnipeds with chronic conditions may be overrepresented due to their ability to haul out onto land compared to fully aquatic cetaceans and non-nesting sea turtles and to those pinnipeds that experience acute morbidity or mortality off shore^14^. Strandings that occur close to human habitation or during the summer when beach tourism peaks introduce potential geographic and seasonal reporting bias, respectively. These are mitigated, however, because the MMSC operates year-round and NJ is densely populated with development along the length of its coastline including municipalities and residences inhabited year-round.

When accounting for all 4,812 cases included in this study, a greater proportion of phocids were presented alive compared to proportions of cetaceans and sea turtles. Among all cases that presented alive, phocids had the highest likelihood of release compared to cetaceans and sea turtles. As in phocids from other populations presented for rehabilitation^22^, it is likely that juveniles usually stranded due to conditions that were more responsive to supportive care and empirical treatment (e.g. starvation, exhaustion, heavy parasite burdens, infectious disease) than adults that stranded due to morbidities that carry a poorer prognosis regardless of intervention. The success of rehabilitation for juveniles may explain the improved chances of release amongst phocids (a higher proportion of which were juveniles compared to other taxa) and for animals presented in winter and spring months which coincide with phocid pupping and weaning. It is more likely for juvenile cetaceans and sea turtles to die at sea and be scavenged, than for juvenile phocids which may naturally seek to haul out on land when ill or injured.

Across all species, fewer adults and neonates presented alive compared to juveniles. Within these age classes, many of the successfully released animals were adult cetaceans and sea turtles. Furthermore, it is possible that successfully released adults frequently stranded due to causes which allowed for release either immediately or after brief observation, compared to juvenile animals which often required a longer period of rehabilitation. Therefore, age class of the patient at time of presentation to MMSC responders did not affect the odds of release.

Cetaceans and sea turtles were more likely than phocids to be presented dead and, when presented alive, less likely than phocids to be released. Qualitative review of stranding records indicates that cetaceans that were released were mostly those which received immediate care at the beach or entangled offshore and less likely to be released after long-term care than sea turtles or pinnipeds. Sea turtles were most likely to present in the summer and autumn, and when presented alive, more likely to be released compared to other outcomes, which may explain the greater odds of release during summer months and similar odds of release in autumn relative to winter and spring.

Sex was not expected to have an effect on the odds of release and released animals had equal odds of being male or female. However, those taxa whose sex was more difficult to determine, cetaceans and sea turtles, were also more likely to strand due to anthropogenic causes such as entanglement, while clinically healthy and receive immediate care and be released. Therefore, animals of unknown sex were most likely to be released.

The proportion of successful releases increased from the first decade the MMSC was in operation, 1976–1986, to the most recent decade, 2007–2016, with similar odds of release in 1987–1996 and 1997–2006 to the most recent decade. Over the duration of the study period, the MMSC has not undergone any management changes and has benefitted from experienced management and staff. The MMSC’s facilities have also expanded in size and capacity for patients requiring long term care. Increased odds of release after the first decade likely reflects these improvements in operations since the MMSC’s inception.

Many marine species migrate and are only seasonally present in NJ coastal waters. The tendency of sea turtles to strand in summer and autumn and of phocids to strand in winter and spring coincide with the presence of these taxa along the NJ coast and are unlikely to reflect a seasonal change in threats as might be expected for year-round inhabitants. During the winter, cetaceans were less likely to be presented than were phocids. This is likely due to the seasonal presence and absence of phocids, as the distribution of cetaceans was fairly even year-round. The cetaceans presented to the MMSC included 23 species of odontocetes and 5 species of mysticetes. Therefore, no single variable is likely to explain temporal stranding patterns for this group.

Sea turtles that presented alive were more likely to be released than to die or be euthanized. This may have been due to live animals stranding due to entanglement. Although evaluation of proximate causes of stranding was outside the scope of this study, this supposition is supported by qualitative review of the stranding records. These records do not suggest that animals stranding due to anthropogenic insults received increased effort for recovery and rehabilitation from MMSC responders than those that stranded due to natural causes. Live phocids and cetaceans were more likely to die under care than to be euthanized. For free-ranging animals presented to rehabilitation centers, humane euthanasia is preferable to natural death in order to limit animal suffering and expenditure of resources on cases that carry grave prognoses. Indications for euthanasia may not be readily recognized in phocids which naturally haul out on land, whereas live stranded cetaceans are often obviously moribund and suffering. For any species, euthanasia may be delayed until a licensed veterinarian trained to work with non-domestic species is present. Humane euthanasia of cetaceans, in particular, is logistically challenging as vasculature is difficult to access, large volumes of drugs are required, and the carcass must be disposed of appropriately to avoid drug residues contaminating the environment or entering the food chain^23^. Exsanguination can be controversial and is avoided especially since euthanasia is often performed on the beach in full view of the public.

Few animals met the criteria for transfer to another facility. These included phocids and sea turtles that were deemed unable to survive and successfully reproduce outside of human care, but were expected to have a good quality of life in an appropriate captive environment and a suitable facility was available at time of rehabilitation. Other phocids, sea turtles, and cetaceans were transferred if release was considered more likely should the animal be rehabilitated at a different institution.

Taxa, age, sex, and season can be used to predict the probability of release of an animal presented to the MMSC across all decades. Decade of presentation is independent of these variables and does not help predict the probability of release when these other variables are considered. The predictive variables of taxa, age, sex, and season, however, are not independent of one another. The taxa investigated occur and reproduce seasonally along the NJ coast. Animals of unknown sex would be more likely to be released as these were more likely to be cetaceans or sea turtles released on site without an examination to determine sex. Most animals that were presented alive were juvenile phocids that occur in NJ only during winter and early spring thus explaining the use of these variables to predict outcome.

## Conclusion

This retrospective study is representative of previously unreported data of cases of marine animals stranded along the entire NJ coastline. Retrospective analysis of data derived from marine animal stranding centers is recommended to critically review the rehabilitation process. The results can generate evidence-based recommendations to minimize animal suffering, improve successful outcomes, efficiently administer resources, and assess the health of free-ranging populations.
